# Novel Patient Cell-Based HTS Assay for Identification of Small Molecules for a Lysosomal Storage Disease

**DOI:** 10.1371/journal.pone.0029504

**Published:** 2011-12-21

**Authors:** Haifeng Geng, Grace Whiteley, Jameson Ribbens, Wei Zheng, Noel Southall, Xin Hu, Juan J. Marugan, Marc Ferrer, Gustavo H. B. Maegawa

**Affiliations:** 1 McKusick-Nathans Institute of Genetic Medicine, Johns Hopkins University School of Medicine, Baltimore, Maryland, United States of America; 2 Department of Pediatrics, Johns Hopkins University School of Medicine, Baltimore, Maryland, United States of America; 3 National Institutes of Health, NIH Chemical Genomics Center, Rockville, Maryland, United States of America; Johns Hopkins School of Medicine, United States of America

## Abstract

Small molecules have been identified as potential therapeutic agents for lysosomal storage diseases (LSDs), inherited metabolic disorders caused by defects in proteins that result in lysosome dysfunctional. Some small molecules function assisting the folding of mutant misfolded lysosomal enzymes that are otherwise degraded in ER-associated degradation. The ultimate result is the enhancement of the residual enzymatic activity of the deficient enzyme. Most of the high throughput screening (HTS) assays developed to identify these molecules are single-target biochemical assays. Here we describe a cell-based assay using patient cell lines to identify small molecules that enhance the residual arylsulfatase A (ASA) activity found in patients with metachromatic leukodystrophy (MLD), a progressive neurodegenerative LSD. In order to generate sufficient cell lines for a large scale HTS, primary cultured fibroblasts from MLD patients were transformed using SV40 large T antigen. These SV40 transformed (SV40t) cells showed to conserve biochemical characteristics of the primary cells. Using a specific colorimetric substrate para-nitrocatechol sulfate (pNCS), detectable ASA residual activity were observed in primary and SV40t fibroblasts from a MLD patient (ASA-I179S) cultured in multi-well plates. A robust fluorescence ASA assay was developed in high-density 1,536-well plates using the traditional colorimetric pNCS substrate, whose product (pNC) acts as “plate fluorescence quencher” in white solid-bottom plates. The quantitative cell-based HTS assay for ASA generated strong statistical parameters when tested against a diverse small molecule collection. This cell-based assay approach can be used for several other LSDs and genetic disorders, especially those that rely on colorimetric substrates which traditionally present low sensitivity for assay-miniaturization. In addition, the quantitative cell-based HTS assay here developed using patient cells creates an opportunity to identify therapeutic small molecules in a disease-cellular environment where potentially disrupted pathways are exposed and available as targets.

## Introduction

In the drug discovery process, the use of cell-based assays for high throughput screening (HTS) has become increasingly common as targets are presented in a more relevant biological context than classical *in vitro* biochemical assays [1]. Cell-based HTS assays also allow the identification of compounds that cross cell membranes, and reach specific cellular compartments, providing simultaneously cell permeability and cytotoxicity information at an earlier stage of the screening [2]. However, in most cellular HTS assays, cells utilized are usually not disease relevant. Thus, the use of patient-derived cell lines for HTS campaign brings considerable advantages when comparing with the traditional single target biochemical assays. Cellular assays using patient cells provide a unique opportunity to assess a target protein and/or pathway in the context of potentially disrupted biochemical and/or signaling pathways secondary to the disease process. In addition, this pathogenic *in cellulo* setting permits the evaluation of multiple intervention points, which are potentially altered in the disease, as opposed to commonly used cell-expression systems of a specific protein target or a predefined step of a purified or recombinant protein-based assay.

Recently, small molecules have become an attractive approach for the treatment of lysosomal storage diseases (LSDs), inherited metabolic disorders resulting from mutations in genes encoding proteins crucial for lysosomal function [3]. The almost 60 LSDs are individually rare, however, collectively, their incidence is about 1/7,000, making these disorders an important health problem worldwide [4]. Despite the important advances in treatment for LSDs, only six have FDA-approved drugs that control or attenuate some of the disease-related symptoms [5]. As most of these drugs are recombinant enzymes that are unable to cross the blood-brain barrier (BBB), only non-neurological symptoms are treated. A considerable number of HTS assays have been developed to identify small molecules (<500 Da), which are more likely to cross the BBB. Some of them function as pharmacological chaperones (PCs), enhancing the levels of deficient misfolded mutant lysosomal enzymes by physical interactions that assist their folding [6], [7], [8]. Most missense mutations encode misfolded lysosomal enzymes that are partially functional as the majority of these mutant proteins are degraded early by the ER-associated degradation (ERAD) pathways [9]. Most of HTS assays developed to identify PCs have been based on single-target biochemical assays [10], [11], [12]. Based upon these observations, a cell-based HTS assay will allow the identification of small molecules that function not only as PCs, but also others that enhance the residual lysosomal enzymatic activity by acting in different molecular pathways that result in increased levels of the mutant, but still partially functional enzyme in the lysosomal compartment [13].

Here we demonstrate the development of a cell-based HTS assay in 1,536-well plates utilizing cultured skin fibroblasts from a patient with metachromatic leukodystrophy (MLD), a LSD caused by deficiency of arylsulfatase A (ASA), resulting in severe demyelination secondary to the accumulation of sulfatide lipid in myelin [14]. No specific treatment is currently available for this neurological LSD. The main goal of the study was to generate a reliable cell-based HTS assay to screen chemical compound collections to identify small molecules with therapeutic potential for MLD. We developed a novel patient cell-based fluorescent HTS assay taking advantage of the ability of para-nitrocatechol (pNC), product of desulfation of a colorimetric substrate for ASA, to quench fluorescence emitted from white solid-bottom multi-well plates. This method increases considerably the sensitivity and statistical robustness of the HTS assay for ASA when testing it against a small compound collection library.

## Results

### Establishment and characterization of SV40-transformed fibroblasts from MLD patients

**Figure 1:**
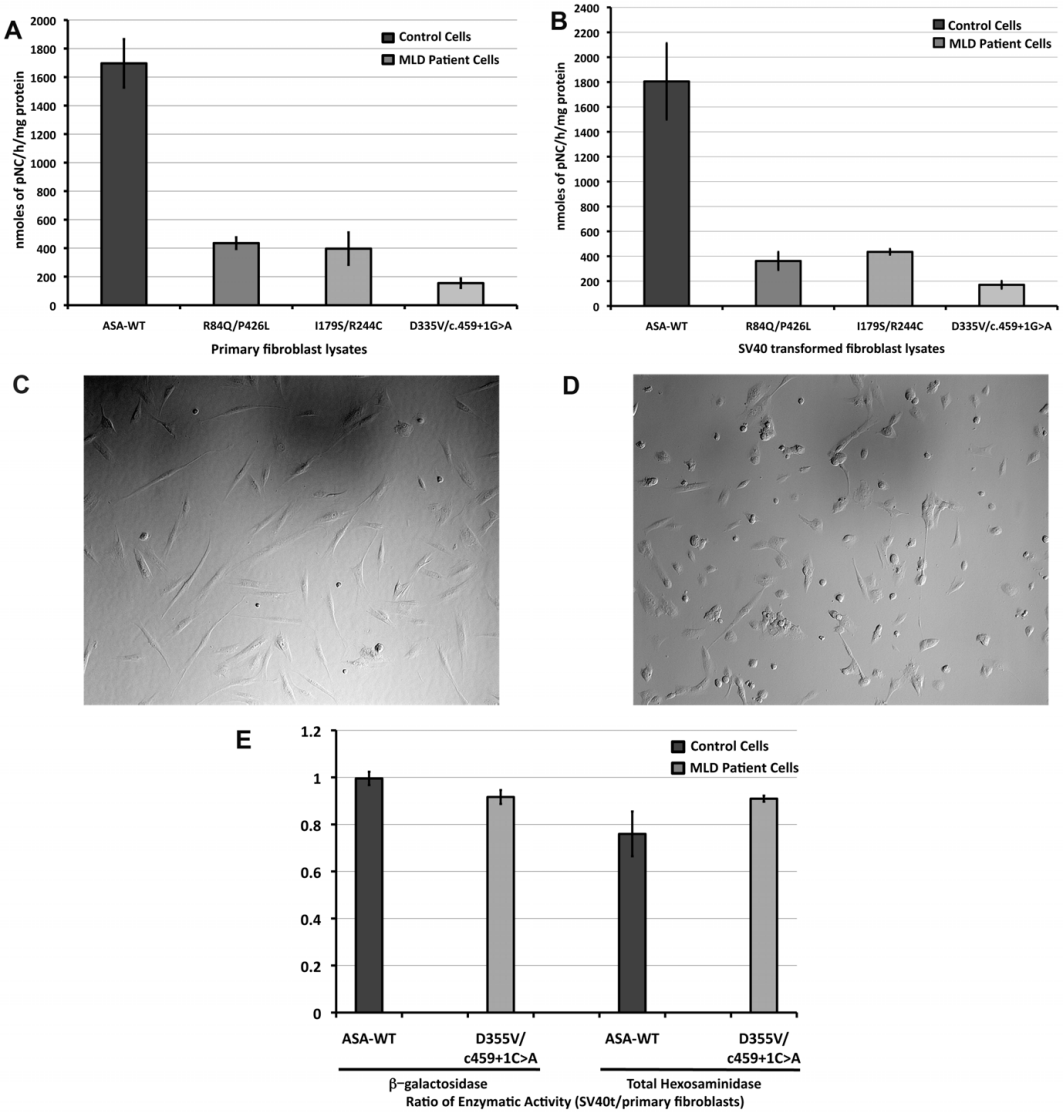
Biochemical and morphological comparison between primary and transformed cultured skin fibroblasts. Primary and SV40-transformed (SV40t) skin fibroblasts were culture in 75 cm^2^ flasks up to confluence before being harvest to obtain cell lysates to perform ASA activity assays. (**A**) ASA enzymatic activity of primary skin fibroblast, control with wild type ASA (ASA-WT) and three MLD patient cell lines with different *ARSA* mutations. Each of these cell lines carries a previously described mutant ASA with a residual enzymatic activity (I179S, P426L, D335V). ASA activity is expressed in nmoles of pNC per hour and standardized by protein concentration. (**B**) ASA activity from SV40t fibroblasts showed comparable ASA activity with primary cell lines. (**C**) Cell morphology of primary skin fibroblasts showed the classical fusiform shape. The SV40t counterparts (**D**) show distinguishable polygonal shape. (**E**) The ratio of enzymatic activity of two other lysosomal enzymes beta-galactosidase and total beta-hexosaminidase are shown. Both the control (ASA-WT; dark gray) and MLD patient cells (ASA-D355V/c.959+1C>A; light gray) showed a ratio close to 1.

Primary skin fibroblasts from patients with several LSDs are one of the most reliable cell lines to study LSDs [15]. Most key biological concepts in LSDs have been discovered in cultured skin fibroblasts from patients with diverse types of these genetic diseases [16], [17]. Cultured skin fibroblasts from patients with MLD were assembled and the *ARSA* gene sequenced to determine mutations and potential polymorphisms that commonly affect the ASA activity [14]. In order to have sufficient cells for the HTS assays, cultured skin fibroblasts were transformed using large T antigen from simian virus (SV40). Primary cells cultured to confluence in 75 cm^2^-flasks, when harvested and re-suspended in a specific medium volume, generated a concentration of 200–300 cells/microL. When SV40t cells were grown to confluence in the same flasks, a higher concentration of 1,000–2,000 cells/microL was observed after culture, trypsinization and res-suspension under the same conditions. The increased cell number of cultured SV40t cell lines is explained by their growth being less inhibited by cell contact, which is consistently observed in cultured primary fibroblasts. In SV40t fibroblasts, ASA enzymatic activity is conserved in both controls with wild type ASA (ASA-WT) and MLD patient cells with mutant ASA that retain residual ASA activity. After SV40 transformation, the difference in ASA activity between controls and MLD patients is also preserved. A comparable level of residual ASA activity is seen in SV40t fibroblasts from patients with late onset forms of MLD (Fig.1A and 1B). Additionally, the morphology of SV40t cells is altered to a typical polygonal shape (Fig.1C), in contrast with the fusiform-shape of primary lines (Fig.1D). The level of other lysosomal enzymes, such as total beta-hexosaminidase (Hex) and beta-galactosidase, from SV40t MLD patient (ASA-D355V/c.495+1C>A) and control (ASA-WT) cells were not significantly different from the levels obtained from the original and corresponding primary cell lines (Fig.1E).

**Figure 2:**
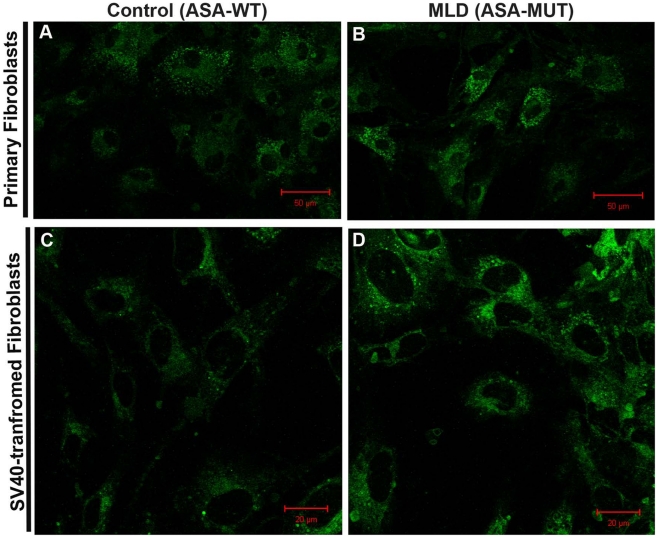
*N*BD-sulfatide assay in primary and SV40 transformed cultured fibroblasts. Primary and SV40-transformed (SV40t) culture fibroblasts from MLD patient (c.459+1G>A/E484L) were exposed over 24hs to 10 nmoles of albumin-conjugated of *N*BD-sulfatide (see *Methods*). The ability of ASA enzymatic activity to degrade the exogenous fluorescent-labeled sulfatide, *N*BD-sulfatide, can distinguish MLD patient primary fibroblasts (ASA-MUT) (**B**) from controls (**A**) with normal ASA-WT enzymatic activity. SV40t fibroblasts from MLD patient (**D**) also demonstrated elevated accumulation of *N*BD-sulfatide when compared with SV40t control fibroblasts with normal ASA activity (**C**).

When treating cells with artificial sulfatide analog (*N*-lissamine rhodaminyl-(12 aminododecanoyl)cerebroside 3-sulfate (*N*BD-sulfatide), conjugated to free lipid albumin [18], increased accumulation of this fluorescent sulfatide, is observed in primary and as well as SV40t MLD patient cell lines in comparison to respective controls (Fig.2). This result indicates that conservation of the impaired degradation of the *N*BD-sulfatide in ASA deficient cells after SV40 transformation.

### Exploring the fluorescence properties of white solid-bottom plates using the classical colorimetric substrate for ASA

**Figure 3:**
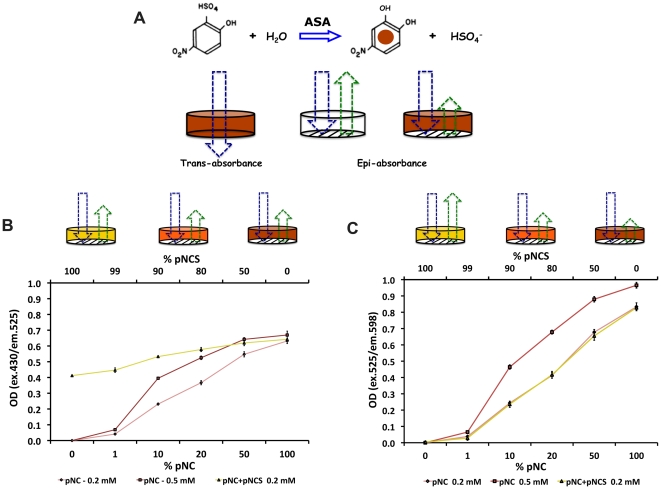
Development of a fluorescence-quench absorbance assay for ASA using a colorimetric substrate in white solid-bottom 1,536-well plates. (**A**) Basis of the fluorescence-quench absorbance assay. The desulfation of pNCS by ASA generates pNC, a colorimetric product, whose optimal absorbance is detectable at 515 nm. Thus, the reaction is performed in clear-bottom (transparent) microplates to measure trans-absorbance. Since white plastic solid-bottom plates have fluorescence properties [22], when an excitation light is directed to white solid-bottom microplates, emission light can be detected by epi-absorbance (**A**). The green arrow represents the fluorescence signal emitted from of solid-bottom of the well of a plate. Therefore, the generated pNC product can be used to quench the emission of the light from the bottom of each well in white solid-bottom plates. Based on this principle, the optimal concentrations of pNCS (yellow well) and pNC (brown well) were tested in white solid-bottom 1,536-well plates. Solutions with increasing %concentrations of 0.2 mM (pink lines) and 0,4 mM (red lines) of pNC were used. A pNC+pNCS mixed solution with decreasing %concentrations of 0.2 mM pNCS substrate and increasing %concentrations of 0.2 mM pNC product was used to simulate the effects of ASA over the pNCS substrate. (**B**) At excitation 430 nm and emission 525 nm (close to pNC absorbance, 515 nm), the pNCS substrate quenches fluorescence from the white solid-bottom plate showing an OD of ∼0.4 at its initial concentration0.2 mM; yellow well with decreased green arrow). Consequently, a flat line with increasing of %concentrations of pNC product was generated (yellow line). (C) However, at excitation 525 nm (close of 515 nm) and emission 598 nm, pNCS no longer quenches the fluorescence from white solid-bottom plate (yellow well with increased green arrow), demonstrating an increasing OD signal with the increase of %pNC (product) and decreased %pNCS (substrate) in the mixed solution of pNCS+pNC. This pNC+pNCS solution (yellow line) overlaps with the solution containing only pNC at 0.2 mM (pink line).

Under specific assays conditions, the classical para-nitrocatechol sulfate (pNCS) colorimetric substrate permits the distinction of ASA activity from arylsulfatase B (ASB), another lysosomal sulfatase [19], [20]. However, the use of a colorimetric substrate with its inherent low sensitivity limits miniaturization of this ASA assay into higher density microplates. Traditionally, colorimetric assays are not readily scalable to small volumes required for miniaturization into high dense plates [21]. A method was described to miniaturize colorimetric assays to 1,536-well format using fluorescence properties of white solid-bottom microplates [22]. In this fluorescence- quench absorbance assay, absorbance is measured by the decrease of fluorescent signal from white material of the plate at an excitation or emission wavelength that matches the absorbance maximum of the colored reaction product. Based on this principle, experiments were performed to determine optimal capturing fluorescence of white solid-bottom 1,536-well plates. The desulfation of pNCS by ASA generates para-nitrocatechol (pNC) product, which has an orange to red color (Fig.3A). Given the fluorescence properties of white solid-bottom 1,536-well plates and the pNC absorbance, quenching of emitted fluorescence by pNC, was tested using increasing concentrations of pNC product at two conditions: excitation 430 nm and emission 525 nm and excitation 525 nm and emission at 598 nm (Figs.3B and 3C). In each of these two assays, the pNC maximal absorbance ∼515–520 nm matched with emission (525 nm; Fig.3B) or excitation (525 nm; Fig.3C). At excitation 430 nm and emission 525 nm, the substrate pNCS showed to quench the fluorescence emitted by white solid-bottom plate. Therefore, less plate fluorescence remains to be quenched by the increasing concentrations of the product pNC and decreasing concentrations of pNCS in a mixed pNCS/pNC solution (Fig.3B). However, using a red-shifted wavelength (525/598 nm - excitation/emission), pNCS (substrate) no longer quenches plate fluorescence, allowing pNC (product) to quench it gradually as its concentrations increases (Fig.3C). The fluorescence reading parameters were set at excitation 525 nm and emission 598 nm in order to obtain the optimal fluorescence quench absorbance with the pNC product generated from ASA reaction against colorimetric substrate pNCS.

### Miniaturization into 384-well plates using SV40 transformed fibroblasts

**Figure 4:**
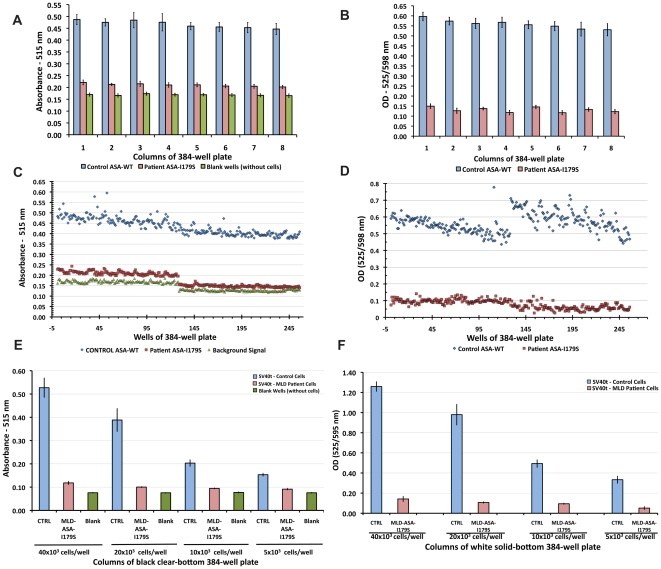
Testing the fluorescence-quench absorbance assay as a throughput assay for ASA activity in cultured cells. (**A**) In cultured SV40t fibroblasts from control (ASA-WT) and MLD patient (ASA-I179S), the absorbance ASA assay (515 nm) was tested and showed robust results in black clear-bottom 384-well plates. Each bar corresponds to the mean signal from 16 wells (one plate column). In 384-well white solid-bottom plates, these cultured SV40t fibroblasts showed comparable results were observed (**B**). Optical density (OD) calculation is described in *Methods* section. Inter-leaved 384-well plate assays also showed similar results for both ASA absorbance (**C**) and fluorescence-quench absorbance (**D)**.** In a 384-well plate,** SV40t fibroblasts from control and a MLD patient were seeded at different numbers per well (**E**). Using the 15 mM of pNCS substrate concentration in the substrate/lysis buffer, seeding 40×10^6^ cells/well showed the best results of spectrophotometric assays. By reducing the number of cells in each well, 20×10^6^, 10×10^6^ and 5×10^6^, correspondent and equivalent reductions of absorbance signals were observed. (**F**) The same pattern of results was noted when culturing the same number of cells in a white solid-bottom 384-well plate. However, the optical density (O.D.) measurements read at excitation/emission pair of 525/595 nm from white solid-bottom plate showed improvement in the assay-window between ASA enzymatic activity from SV40t control and MLD patient fibroblasts.

The objective of developing a cell-based HTS assay for ASA is to treat cultured fibroblasts from a MLD patient with a small molecule collection for a specific time period, and subsequently to measure the resultant effect on the residual ASA activity. Preliminary experiments done using primary fibroblasts cultured in 96-well plates demonstrated an encouraging assay-window between controls (ASA-WT) and a selected MLD patient fibroblast cell line (ASA-I179S), which shows the highest ASA residual activity (Supporting Information S1 and Fig.S1). SV40t fibroblasts from both controls and the MDL patient generate higher absorbance signals than primary fibroblasts (Fig.S2). SV40t cells were then cultured in white solid-bottom 384-well plates, assayed with pNCS substrate and fluorescence was measured at excitation/emission pair 525/598 nm in a multiple-well plate reader. The absolute value obtained was then translated into optical density (OD), calculated as OD = −log(*sample/blank*), where *sample* is the signal obtained from a well containing cells (control or MLD cells), culture medium, pNCS substrate buffer and stop reaction solution (assay reagents); *blank* is the mean from signals from wells containing no cells, only cultured media and assay reagents (Fig.4). Blank references generate higher fluorescence signals (405.7±9.5), as no colorimetric product (pNC) is formed from the desulfation of pNCS. Lower fluorescent signals (105.5±15.8) were observed from wells with control SV40t (ASA-WT) cells as they produce more colorimetric pNC product that quenches fluorescence emitted from the bottom of the plate. Cultured SV40t MLD patient fibroblasts (ASA-I179S) generate low levels of pNC product (due to ASA residual activity), resulting in elevated fluorescent signals (354.1±13.7), but still lower than blank reference. The calculated OD increased the assay-window between control (ASA-WT) and MLD patient (ASA-I179S) fibroblasts (Fig.4B and 4D). A “sister plate” was prepared in black clear-bottom 384-well plate to monitor cell status of confluence and measure absorbance (Fig.4A and C). In order to evaluate the ideal cell number to seed in each well, different cell numbers were placed into columns of a 384-well plate. Seeding approximately 40×10^6^ cells/well, the highest absorbance signals in both control (ASA-WT) and MLD patient (ASA-I179S) cells were obtained. As in previous assays, ASA assays performed in white solid-bottom 384-well plates with a fluorescence reading (525/598 nm for excitation/emission) showed a clear and significant difference between SV40t controls and MLD patient fibroblasts (Fig.4E and 4F).

### 1,536-well miniaturization and pilot screening assay against a small drug collection

**Figure 5:**
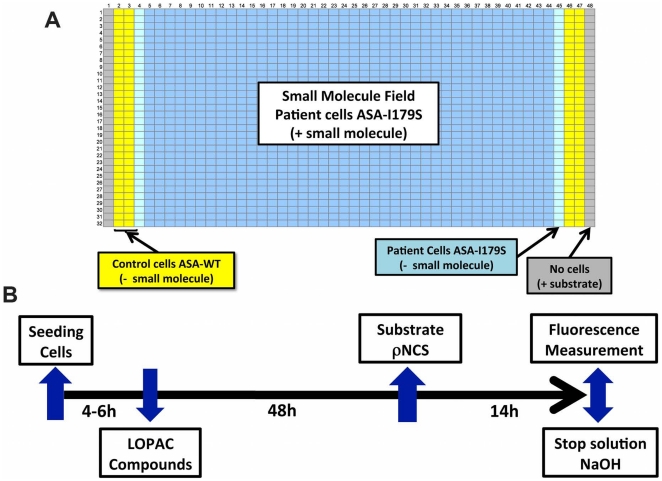
The design of a cell-based HTS assay for ASA in 1,536-well plates. (**A**) The disposition of SV40t fibroblasts from MLD patient (ASA-I179S - blue) and from control (ASA-WT - yellow) in a 1,536-well plate. (**B**) The time-line of events of this cell-based HTS assay for ASA. A library of 1,280 small-molecules at seven different concentrations from 7×10^−3^ to 114.2 microM was used to perform a pilot HTS assay. A total of eight 1,536-well plates in the same format were assayed including one plate with cells treated with DMSO (solvent of compounds) and seven other plates for each of the LOPAC concentration tested.

**Figure 6:**
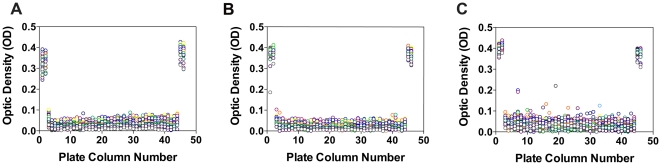
Scatter plot from the quantitative cell-based HTS assay for ASA using the LOPAC library. In each panel, columns 2, 3, 46 and 47 depict OD values from wells with control cells (ASA-WT), which were not exposed to small molecules. OD values from MLD patient fibroblasts (ASA-I179S) treated only with DMSO were located in columns 4 and 45. SV40t MLD patient fibroblasts were seeded in columns 5 to 44. The 1,536-well plates treated with DMSO (0.57%) (**A**), lowest (7×10^−3^; **B**) and highest (114.2 microM; **C**) concentrations of LOPAC are shown. Small molecules, demonstrating increased ODs in the plot C, were studied further in the curve analysis.

Based on the ASA assay parameters developed in 384-well plates, initial experiments were done in 1,536-well plates. In these latter plates, on average 8×10^3^ cells were seeded per well. Table 1 describes the optimal conditions for the ASA assay in 1,536-well plates. In order to test the robustness of the cell-based HTS assay, a pilot screening was performed utilizing the Library of Pharmacologically Active Compounds (LOPAC) containing 1,280 small molecules. The design and time-line of the HTS assay is depicted in figure 5. In the HTS assay, only SV40t cells from MLD patient (ASA-I179S) and control (ASA-WT) were used. In each 1,536-well plate, a control cell line (ASA-WT) was seeded in columns 2, 3, 46 and 47, as no pharmacological chaperone exists for ASA to be used as a positive control for the HTS assay. Columns 4 and 45 contain MLD patient cells only treated with DMSO. Columns 5 to 44 of these high-density plates is known as the “compound area”, which includes 1,280 wells with cultured MLD (ASA-I179S) patient cells (Fig.5B). Thus, one 1,536-well plate accommodates the entire LOPAC library at one small molecule/well (Fig.5A). Using seven 1,536-well plates, each with different concentrations of the LOPAC compounds (7×10^−3^, 36×10^−3^, 0.18, 0.92, 4.5, 22.8 and 114.2 microM), and an additional plate treated only DMSO (solvent of LOPAC compounds), cells were treated for 48 h prior to performing the ASA assay (Fig.6A and 6B). The concentrations used were based on earlier quantitative HTS assays [23]. False-positives were defined as OD signals ≥3 S.D. above the OD mean obtained from the wells containing MLD patient cells. No false-positives were observed in the plate treated only with DMSO (Fig.6A). The coefficients of variance (CVs), Z' factors, signal-to-background ratios of ASA activity (control/MLD patient cells) are shown in Table 2. Figure 6 shows the scattered plot result from the plate treated only with DMSO, the plates treated with the lowest (7×10^−3^ microM) and highest (114.2 microM) LOPAC concentrations. The scatter plot results from the remaining five concentrations of LOPAC (0.92, 4.5, 22.8 and 114.2 microM) are shown in Figure S2.

**Table 1:** Cell-based HTS assay for ASA protocol.

Step	Action	Volume	Time in Total Assay or Timeframe	Description
***1***	Seeding cells	4 microL	0	SV40t cell lines from MLD patient and control are seeded in 1,536-well plate
***2***	Cell Incubation	-	6^th^ hour	37°C and 5%CO_2_
***3***	Pin dispense	46 nL	∼6 h	LOPAC compound plates in DMSO
***4***	LOPAC Treatment	-	48 h	37°C and 5%CO_2_
***5***	ASA assay	4 microL	54^th^ hour	Substrate/lysis buffer – pNCS(1 mM)/TX-100(1%) – final [pNCS] = 0.5 mM
***6***	Substrate Incubation	-	14 h	ASA assay incubation at room temperature
***7***	Dispense	2 microL	68^th^ hour	Stop solution – NaOH (5N)
***8***	Fluorescent Readout	-	68^th^ hour	CCD-based Viewlux reader

ASA, arylsulfatase A; CCD, charge-coupled device, HTS, high throughput screen; NaOH, sodium hydroxide; LOPAC, library of pharmacologically active compounds; MLD, metachromatic leukodystrophy; pNCS, p-nitrocatechol sulfate; SV40t, SV-40 transformed.

**Table 2:** Statistical parameters of the quantitative cell-based HTS for ASA.

LOPAC concentration (microM)	CV (%)		Z' Factor	Signal/background
	Control	MLD		
**DMSO**	1.03	1.94	0.45	12.9±2.2
**7×10^−3^**	0.61	2.58	0.64	20.0±1.5
**36×10^−3^**	0.42	2.66	0.70	20.0±1.1
**0.18**	0.50	1.31	0.68	11.4±1.4
**0.9**	0.48	1.97	0.69	16.1±1.2
**4.5**	0.39	4.49	0.70	30.8±0.8
**22.8**	0.59	2.26	0.57	13.2±1.0
**114.2**	0.58	4.54	0.54	20.1±0.81

ASA, arylsulfatase A; CV, coefficient of variance; DMSO, dimethyl sulfoxide; HTS, high throughput screen; LOPAC, Library of Pharmacologically Active Compounds; MLD, metachromatic leukodystrophy: SD, standard deviation.

### Concentration-response curve analysis of cell-based HTS for ASA

**Figure 7:**
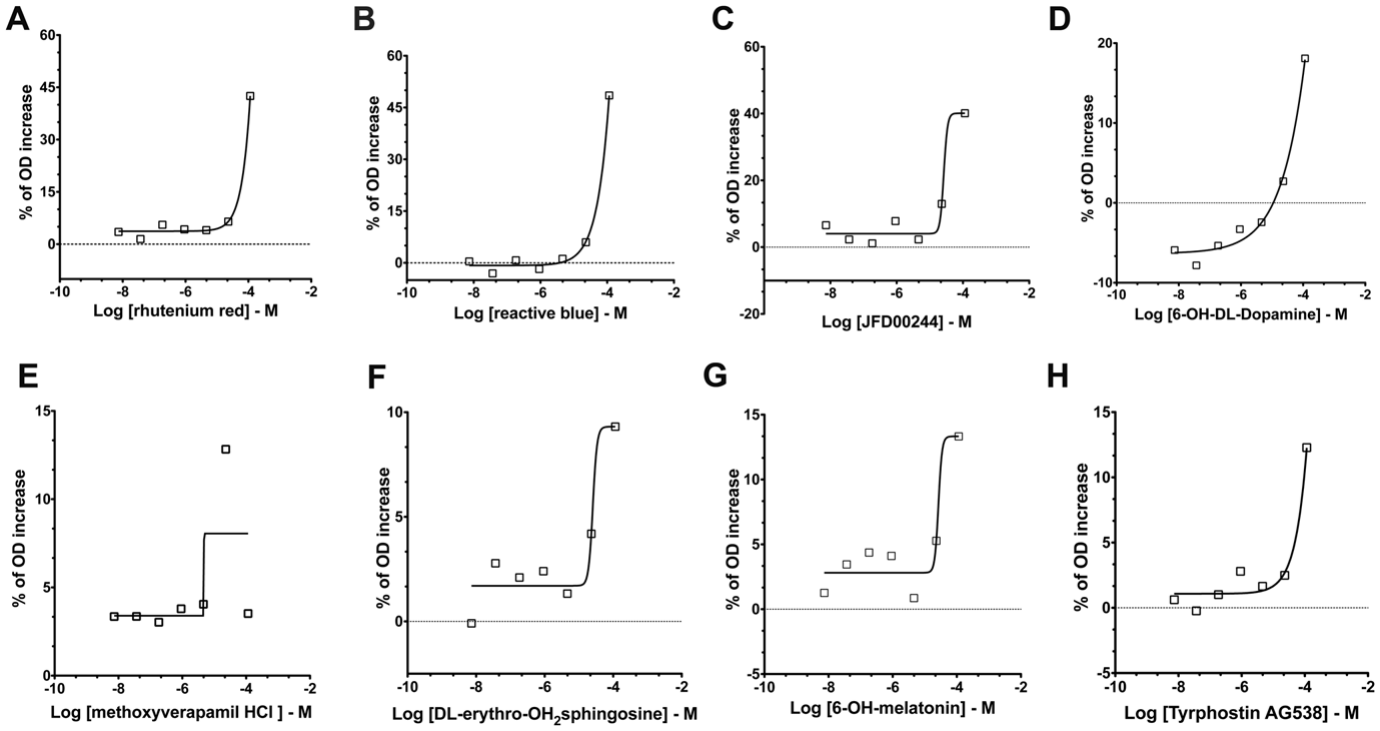
Concentration-response curve analysis obtained from the quantitative cell-based HTS assay for ASA against LOPAC library. In the cell-based HTS for ASA using LOPAC concentrations ranging 7×10^−3^ - 114.2 microM, two compounds, ruthenium red (**A**) and reactive blue (**B**), showed concentration-response curve of class 2.2 (partial curve; *r^2^*≥0.9; efficacy≤80%). Other two small molecules, JFD00244 (**C**) and 6-OH-DL-Dopamine (**D**) also presented concentration-response curves of class 2.2. Four small molecules, methoxyverapamil HCl (**E**), DL-erythro-dihydrosphingosine (**F**), 6-OH-melatonin (**G**) and I-Me-Tyrphostin AG 538 (**H**) showed concentration-response curves of class 2.4 (partial curve; *r^2^*≤0.9; efficacy Min. −80%).

**Figure 8:**
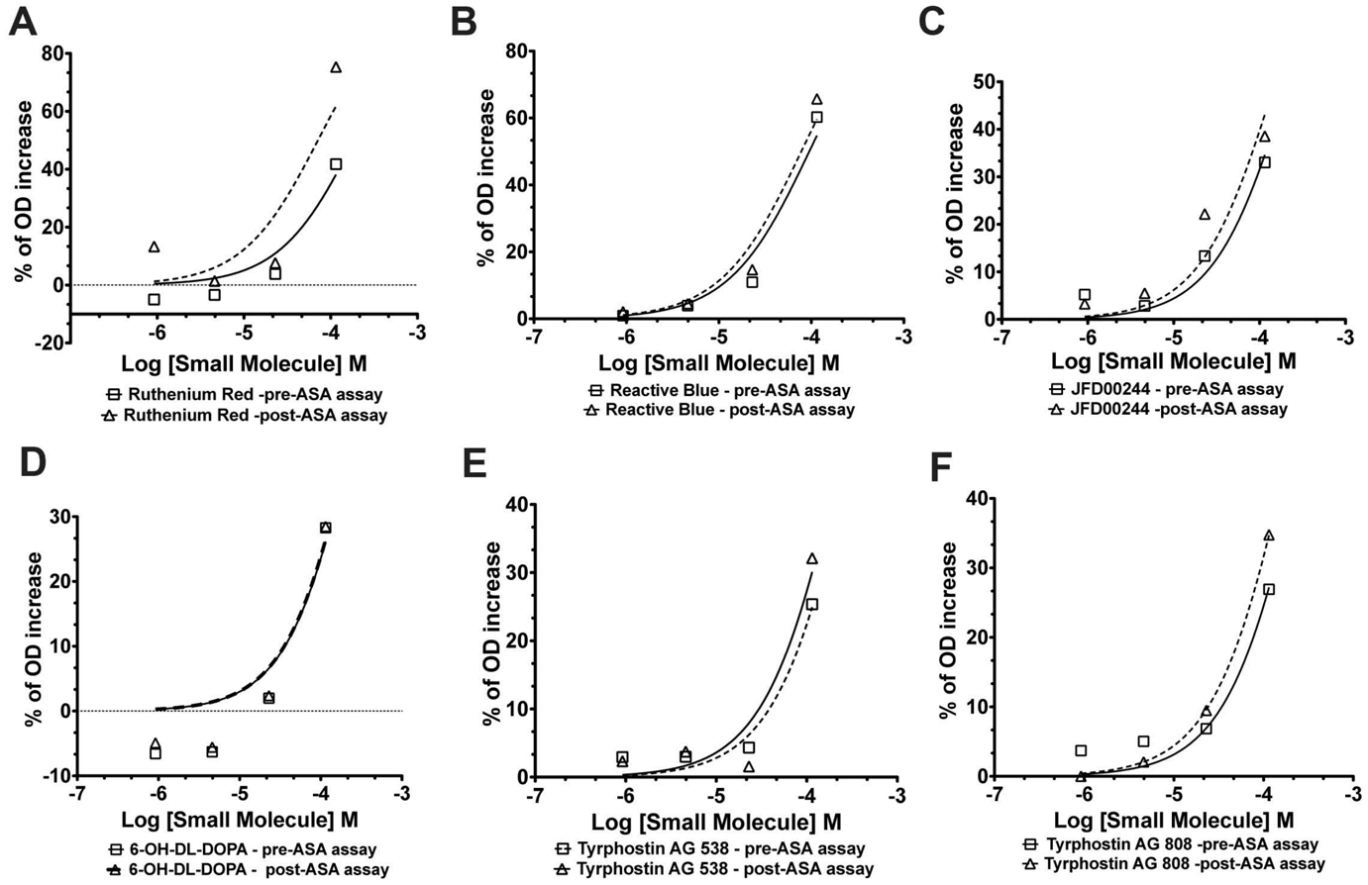
Concentration-response curve analysis of HTS assay for ASA to identify fluorescence and colorimetric compounds. In a 1,536-well plate with the SV40t cultured cells were positioned in the same format as previous assays. LOPAC library was dispensed prior to add substrate/lysis buffer for ASA and after terminating the assay with stop reaction solution. Six compounds depicted in these panels showed increases of the percentage of OD signal (relative to baseline mean of OD value from MLD patient cells). Four highest concentration of LOPAC (0.92, 4.5, 22.8 and 114.2 microM) were used are shown in log scale (in M). Full lines and squares points represent results from the assay in which LOPAC was added just prior to starting ASA assay reaction (pre-ASA assay). Dotted lines and open triangles showed results from the assay in which LOPAC was added after stopping ASA assay reaction (post-ASA assay).

In the developed cell-based quantitative HTS assay for ASA, increases in the OD signals can represent enhancements of residual ASA enzymatic activity in MLD cultured cells (ASA-I179S). Based on the quantitative nature of the screen, in which seven different concentrations of the LOPAC were tested, the selection of candidate compounds is based on the concentration response-curves [23]. The classification of the concentration-response curves was described earlier (Table S1) [23]. The results showed a selective and low “hit” rate in the developed HTS assay. In the concentration-curve analysis, four candidate compounds showed best concentration-response curves classified as class 2.2, which are defined as “partial curves” (containing one asymptote) with *r^2^*≥0.9 and efficiency <80% (% OD enhancement) [23] (Table S1 and Fig.7A–D). Two candidate compounds were fluorescent dyes: ruthenium red, which absorbs light at ∼533 nm and reactive blue, which absorbs at 605 nm (Fig.7A and 7B). Since in this HTS assay for ASA, fluorescence is measured at excitation 525 nm and emission 595 nm, these two compounds are quenching either excitation (ruthenium red) or emission (reactive blue) used for the HTS assay. Therefore, the absorption and emission wavelengths of the two compounds explain the reason why they were identified as candidates. Two other candidate small molecules also showed concentration-response curves of class 2.2 (Fig.7C and 7D). One of them is also colorimetric compound (JFD00244). In order to exclude the potential fluorescent and colorimetric small molecules, two additional HTS assays were performed: one adding the LOPAC compounds just before dispensing the substrate/lysis buffer (pre-ASA assay) and another adding LOPAC compounds after stopping the assay reaction (post-ASA assay). These HTS were performed in 1,536-well plates with the same disposition of SV40t cells. The four highest concentrations of LOPAC (0.92, 4.5, 22.8 and 114.2 microM) were used to identify colorimetric and fluorescent compounds (Fig.8). These two assays were confirmatory and revealed that the color small molecules ruthenium red, reactive blue, JFD00244, 6-OH-DL-Dopamine (curve class 2.2) present increases of OD values correlating with increases of their concentrations (Fig.8A–D). Other compounds including the tyrphostin AG 538 and AG 808 (curve class 2.4) also showed the same pattern of increases of OD values (Fig.8E and 8F).

**Figure 9:**
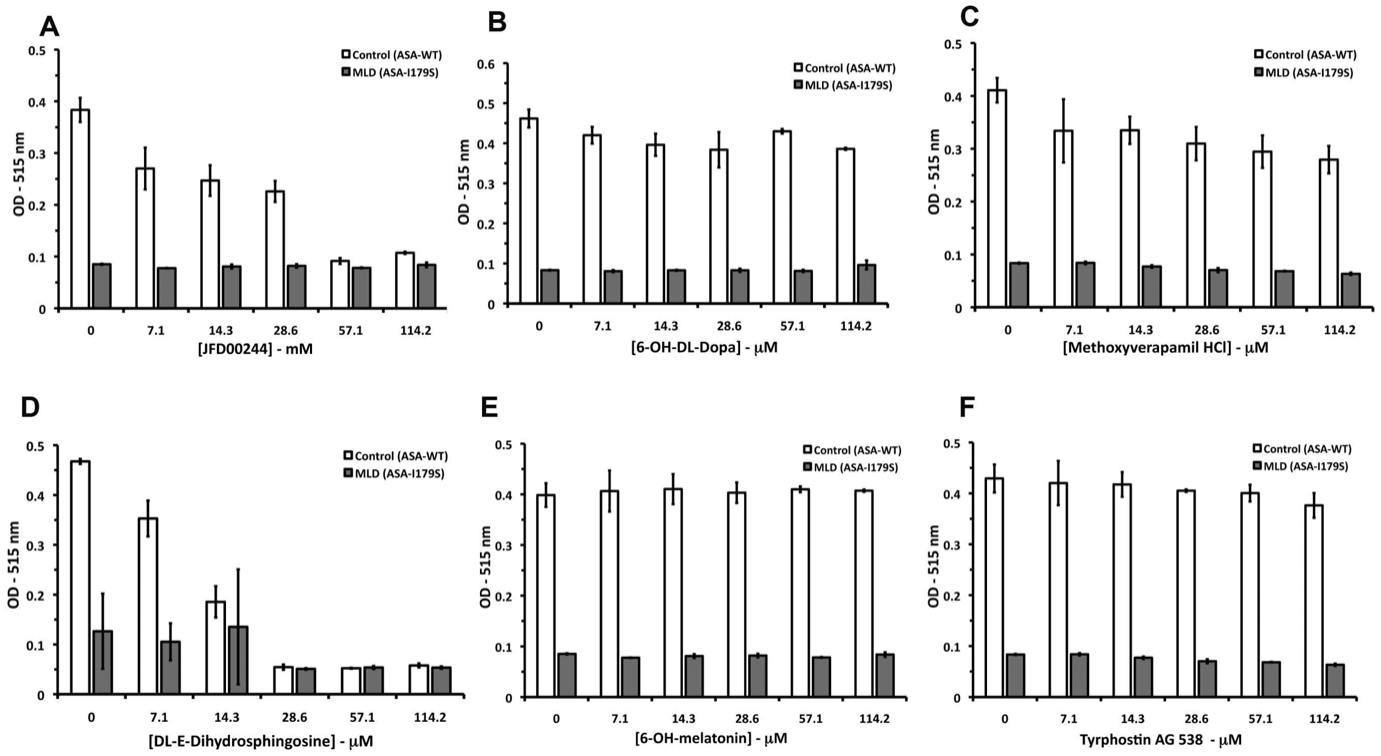
Characterization of candidate compounds in primary culture MLD patient and control cells. The six compounds with concentration-response curves of classes 2.2 and 2.4 were used to treat primary MLD patient (ASA-I179S) and control cells (ASA-WT). The small molecule concentrations used in this assay were within those used in the primary screening. No significant enhancement of residual ASA-I179S enzymatic activity in MLD patient primary fibroblasts was observed. The small molecules tested where those with concentration-response curve of class 2.2: JFD00244 (**A**); 6-OH-DL-Dopa (**B**) and class 2.4: methoxyverapamil HCl (**C**); DL-E-dihydrosphingosine (**D**), 6-OH-melatonin (**E**) and I-Me-Tyrphostin AG 538 (**F**). Reduction of ASA activity in both controls (ASA-WT) and MLD patient fibroblasts (ASA-I179S) were noted as increased concentrations of JFD00244 (**A**), DL-E-dihydrosphingosine (**D**) and methoxyverapamil HCl (**C**).

A few other small molecules showed concentration-curves (curve class 2.4) with less optimal fitting (*r^2^*≤0.9) and lower efficiency (Min. − 80%) (Fig.7E–H). In the curve analysis HTS, these small molecules generated subtle increases of OD values (10–15% from mean OD) (Fig.7D–H). When tested in primary cultured fibroblasts from MLD patient (ASA-I179S) and controls (ASA-WT), these small molecules with curve class 2.4 failed to produce any significant enhancement of ASA activity in primary control (ASA-WT) and MLD patient (ASA-I179S) fibroblasts (Fig.9C–F). Some of these compounds promoted significant reduction of ASA activity in both control and MLD patient fibroblasts (Fig.9A, 9D and 9C). Cell viability assays confirmed the cytotoxicity of these small molecules (Table S2).

## Discussion

**Figure 10:**
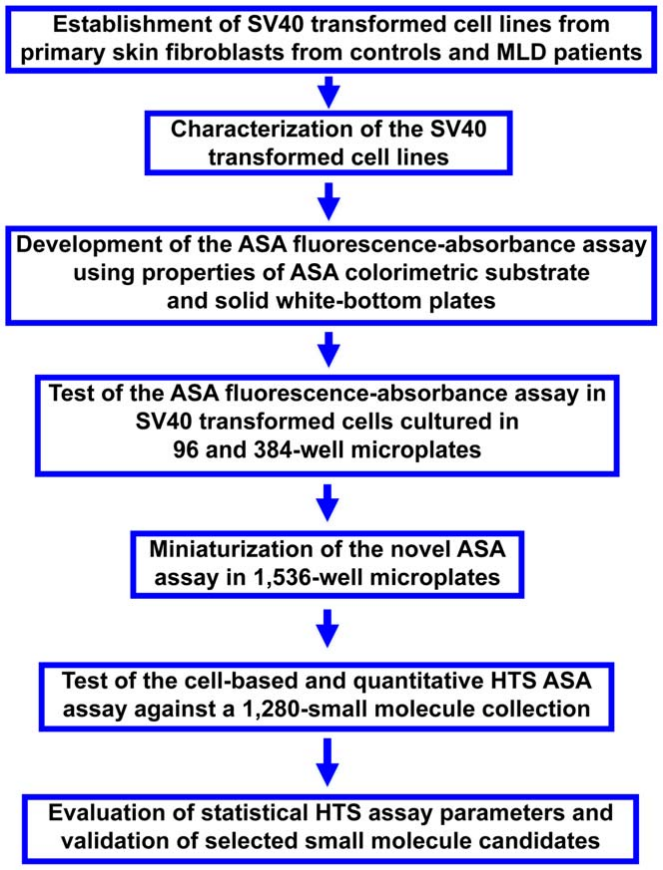
Algorithm summarizing the developed patient cell-based HTS assay for ASA.

Here we report a patient cell-based HTS assay for the identification of potential drug candidates for a LSD. The utilization of a representative patient cell line already in the primary stage of HTS offers considerable advantages in the discovery of small molecules of potential disease relevance. The use of patient cells displays a “disease cellular environment” where numerous disrupted molecular pathways secondary to the primary biochemical/genetic defect are present. These secondary pathogenic cascades have been shown to occur and have implications in the molecular mechanism of several LSDs [24]. Several cell-based assays have been developed for HTS including second messenger mobilization after GPCR activation [25], reporter gene assays [26] and phenotypic assays for cellular processes (*e.g.,* cell migration [27], cytokinesis [28]). However, none of the previously described HTS assays utilized patient-derived cells. Another advantage of patient cell-based HTS assays is that several potentially altered molecular pathways can be exposed to the tens or hundred thousands of small molecules from a specific library. Therefore, novel molecular pathways that are indirectly interacting with the target protein and/or pathway of interest are amenable to be identified and subsequently used as therapeutic targets. An algorithm summarizing the development of the HTS assay here described is depicted in figure 10.

To have access to sufficient number of cells for a large-scale HTS assay, the transformation using pSV3 plasmid carrying the SV40 large T-antigen was required. The SV40t skin fibroblasts from MLD patients conserve the deficiency of ASA as observed in transformation of primary fibroblasts from other LSDs [29]. The SV40t fibroblasts maintain the same level of ASA activity along with other lysosomal enzymes as observed in primary cells (Fig.1). In cultured primary fibroblasts, patients with late-onset forms of MLD have up to 10% of residual ASA activity [30]. SV40t fibroblasts from MLD patients present consistently the same residual ASA activity levels when compared to SV40t control cells with ASA-WT activity (Fig.1). Since pseudo-polymorphism alleles (p.1049A>G; p.96A>G) are common in different ethnic groups [31] resulting in lower ASA activity, *ARSA* genes of controls were also sequenced and confirmed wild-type *ARSA* sequence. The SV40t cells have different morphology from spindle-shaped form of primary cells as predicted (Fig.1). As demonstrated in primary cultured fibroblasts, SV40t MLD patient cell lines also present elevated accumulation of the fluorescent *N*BD-sulfatide after 24 hs exposure in comparison to correspondent SV40t controls (Fig.2).

The ultimate goal of the cell-based HTS for ASA is to identify small molecules that enhance the residual ASA activity found patients with MLD. Therefore, the readout the HTS assay is based on detectable residual ASA activity in MLD cultured fibroblasts. The candidate small molecules are predicted to interact directly with the mutant ASA, functioning as PCs, *i.e.* binding to the misfolded ASA and assisting its folding [9]. Since the developed HTS assay is a cell-based assay, other classes of small molecules that interact with different elements of molecular pathways involved in the folding, trafficking, maturation or degradation of the misfolded ASA mutant resulting in enhancements of its residual activity, will also be identified. During the throughput assay development, the use of pNCS colorimetric substrate detected measurable residual ASA activity from the MLD patient cells with ASA-I179S. This specific MLD patient cell line was chosen, as it is one of the most frequent *ARSA* mutations with the highest residual ASA activity (Fig.1 and 3) [32]. Approximately 80 missense mutations have been identified in MLD patients with residual ASA catalytic activity. About half of the missense mutations lead to arrest of the mutant protein in the ER due to misfolding [33]. Initially, assay conditions adjusting the optimal substrate concentration were developed in 96-and 384-well plates (Fig.S1). In order to streamline the protocol, the step of media removal was eliminated and consequently the use of non-phenol red cultured medium with substrate buffer containing non-ionic detergent was used to assure cell lysis (Fig.3).

The miniaturization of the assay into small volumes (<10 microL) is required for high-dense microplates. In this setting, fluorescence and luminescence assays are preferable given their higher signals and consequently superior sensitivity [34], [35], [36]. The colorimetric substrate pNCS produced robust data up to 384-well plates (Fig.4A and 4C). In order to overcome the limitation of the low sensitivity of the colorimetric substrate, the fluorescent properties from white solid-bottom polystyrene plates and the characteristics of pNC product were explored. In this study, the colorimetric product pNC (generated from ASA desulfation of pNCS) quenches the intrinsic fluorescence of white solid-bottom plate at a specific emission and excitation pair 525/598 nm. This ability of the ASA product pNC to quench the white plate fluorescence allows the use of epi-absorbance mode to measure the ASA activity of SV40t MLD patient and control fibroblasts cultured in these white solid-bottom plates (Fig. 3A). In preliminary studies in 384-well plates, higher fluorescence signals were obtained from blank wells (no pNC product generated) in comparison to fluorescence signals from wells with control (significant pNC product generated by ASA-WT) and MLD patient fibroblasts (small pNC product generated by residual ASA-I179S). The resultant OD ratio generates higher control/patient ratios than those obtained from the colorimetric absorbance assay (Fig.4B and 4D). When miniaturized to white solid-bottom 1,536-well plates, adapting similar conditions developed in 384-well plates, the fluorescence-quench absorbance for ASA assay showed consistent results.

In order to test the robustness of the cell-based HTS assay using an MLD patient line, a small and structurally diverse chemical compound library was chosen for the pilot screening. LOPAC contains representatives of major target classes of small molecules, which are all <500 Da and solubilized in DMSO. LOPAC is often used to determine “hit” rates and statistical parameters of HTS assays, before embarking on screening larger libraries. The results from the LOPAC pilot-screen revealed the robustness of the MLD patient cell-based HTS assay as measured by Z' scores >0.5 (Table 2). The main reason to perform a quantitative HTS with LOPAC library was to select small molecules that function as enzyme enhancement agents (EEAs) by the pattern of concentration-response curves. In the pilot HTS assay, in which compounds were exposed to cells for 48 h, only four compounds demonstrated concentration-curves fitting in class 2.2 (partial curve; *r^2^*≥0.9; efficacy≤80%) according to previously established criteria for concentration-response curve analysis (Table S2) [23]. These compounds were found to be fluorescent dyes: ruthenium red (∼533 nm) and reactive blue (∼605 nm). Two additional compounds were also shown to be class 2.2 (Fig.7C and 7D). Interestingly, the similar HTS assay, in which LOPAC was added at the highest tested concentrations pre- and post-dispensing the substrate/lysis buffer, was instrumental to screen for these and other colorimetric and fluorescent compounds (Fig.7). Thus, in addition to use the red-shifted wavelength [37], the assay in which the small molecule library is dispensed post-ASA assay (Fig. 8) will be crucial to exclude small molecules that produce OD increases because of their fluorescent or colorimetric properties. At primary screening, other compounds showed concentration-response curves of class 2.4 (partial curve; *r^2^*≤0.9; efficacy Min-80%) with mild enhancements of OD values (10–15%) (Fig.7E–H). Testing these candidate compounds in primary cell lines from controls and MLD patient (ASA-I179S) using the classical absorbance ASA assay was essential to examine their effect on ASA activity and exclude them from further studies (Fig.9). Thus, class 2.4 candidate compounds, which present partial concentration-response curves with poor fit and low efficacy, are unlikely to be regarded as potential “hits” in the developed HTS assay [23].

LOPAC was used to test the statistical robustness of the MLD patient cell-based HTS assay for ASA. Three characteristics of this HTS assay can explain the low “hit” rate of the HTS. First, the cellular nature of the HTS assay, in which compounds with poor cell permeability or cytotoxicity within the concentrations tested, are eliminated already in the primary screening. Since the selection of “hits” is based on detection of enhancements of residual ASA-I179S activity in MLD patient cells, compounds that are cytotoxic will present lower ASA activity and, consequently, no concentration-response curves. In fact, two small molecules showed concentration-response curves of class 2.4 due to their fluorescent properties. In the validation assays, these compounds reduced substantially the ASA activity of primary cells from controls and MLD patient (Fig.9A and 9D). These compounds were then shown to reduce cell viability in cytotoxicity assays (Table S2). Second, the quantitative aspect of the cell-based HTS assay allows selecting and clustering candidate small molecules based on the hierarchical classification of concentration-response curves already in the primary screening. In previous studies, small molecules “hits” from primary screening assays were only characterized as potential EEAs when tested in patient's cultured fibroblasts at different concentrations [6], [7]. Third, using a longer wavelength (>550 nm), as in the developed HTS assay, reduction of the percentage of false-positives from small molecules carrying native fluorescence is observed [37], [38].

In summary, this study demonstrates that cell lines from patients with a LSD can be used in a cell-based HTS assay against small molecule collections (Fig.10). The SV40t fibroblast line from a patient MLD conserved the relative residual ASA activity as observed in primary cells from which they were derived from. Taking advantage of the fluorescence properties of white solid-bottom plates, a fluorescence-quench absorbance ASA assay was developed using the robust and reliable colorimetric pNCS substrate, whose product (pNC) acted as a “plate fluorescence quencher”. The implementation of the developed cell-based HTS assay against larger and diverse small molecule libraries should allow the identification of novel potential drugs for MLD, a devastating neurodegenerative LSDs. This approach can be used for several other LSDs and other genetic disorders, especially those that rely on a colorimetric substrate that limits miniaturization into high-density microplates. The patient cell-based and quantitative HTS assay here proposed creates a novel opportunity to identify numerous small molecules, as several potential therapeutic targets are accessible in this disease-cellular environment.

## Materials and Methods

### Cells, chemical reagents and equipments

Skin fibroblast lines from patients with MLD were obtained from the cell bank at Kennedy Krieger Institute, Johns Hopkins Medical Institutions. Written consents and assents approved by Office of Human Subjects Research, Institutional Review Boards Committee JHM-IRB 2 at Johns Hopkins University School of Medicine were obtained. The study reported was also approved by Institutional Review Boards Committee JHM-IRB X at Johns Hopkins University School of Medicine. Synthetic substrate para-nitrocatechol sulfate (pNCS) and standard p-nitrocatechol (pNC) were purchased from Sigma-Aldrich Inc. and used for the ASA assays. The following fluorescent substrates, all purchased from Sigma-Aldrich Inc., 4-methylumbelliferyl-beta-D-galactopyranoside (MUbetaGal) and 4-methylumbelliferyl-(2-acetamido-2-deoxy)-beta-D-glucopyranoside (MUG) were used to assay beta-galactosidase and total beta-hexosaminidase, respectively. Chemicals including sodium acetate, sodium chloride, sodium hydroxide, sodium pyrophosphate (Na_2_P_2_O_7_), citric acid, sodium phosphate dihydrate, Triton-X100 and human serum albumin were all purchased by Sigma-Aldrich Inc. The protein assay kit (Bradford assays) and protease inhibitor cocktail were purchased from Thermo-Fisher Inc. *N*-Dodecanoyl-(*N*-lissamine rhodaminyl-(12 aminododecanoyl)cerebroside 3-sulfate (*N*BD-sulfatide) used for exogenous substrate cellular assays (Fig.2) was purchased from Matreya Inc. Molecular kits for PCRs and reverse transcriptase-PCRs were purchased from Invitrogen Inc. Specific oligonucleotides for sequencing of *ARSA* genomic and cDNA were designed as previously described [39] and synthesized and purchased from Sigma-Aldrich and IDT Corp. The Library of Pharmacological Active Compounds (LOPAC) used in the pilot screening was received from Sigma-Aldrich Inc. LOPAC was diluted in dimethyl sulfoxide (DMSO). The following microplates were used in ASA assays: (i) 96-well transparent, cell culture treated plates (BD Comp.); (ii) 384-well white solid-bottom, polystyrene, tissue culture treated, flat and solid bottom plates (Greiner CellStar®); (iii) 384-well black polystyrene, tissue culture treated, flat and transparent bottom plates (Greiner CellStar®). The white solid-bottom and black transparent-bottom 1,536-well plates (tissue culture treated with flat bottom) were purchased from Greiner Bio-One. The multi-well plate readers used were SpectraMAX 190 (absorbance for 96-well plates) and SpectraMAX Gemini XS (fluorescence for 96- and 384-well plates) both from Molecular Devices™. Safire^2^ (TECAN) mono-chromator based plate reader was used for absorbance reading from 384-well plate. The pilot screening with 1,536-well plate was performed using the following equipments: Thermo Scientific MultiDrop Combi - to seed cells into the plates; Perkin Elmer 1430 ultraHTS Wallac Microplate Imager ViewLux - for fluorescence reading in 1,536-well plate.

### General Tissue Culture Conditions

Primary and transformed fibroblasts were cultured using Dulbecco's Modified Eagle Medium (DMEM; Medtech Inc.) with and without phenol red containing 10% fetal calf serum (FCS; Gemini Biologicals Inc.). No antibiotics were used in routine tissue culture procedures. Cells were cultured using traditional tissue culture incubators with 5% CO_2_ at 37°C. Cells were washed at least twice in phosphate-saline buffer (PBS) before being harvest. Cell lysates for ASA assays were obtained by re-suspending cells in sodium acetate buffer (0.5 M) with protease inhibitors cocktail (Thermo Fisher). Glycerol at 5% was added, as cells were lysate by freeze-thaw method (∼5 times) without the use of detergents. Trypsin PBS solution (0.05%) was used to dissociate cells. Primary and SV40 transformed fibroblast were cultured in 75 cm^2^ flasks to prepare cultured cells into the different microplates used in the described assays.

### Transformation of primary skin fibroblasts with SV40 large T antigen

Cultured primary skin fibroblasts were transformed using pSV3-neo plasmid containing the simian virus (SV) 40 large T cell antigen. The plasmid pSV3-neo (ATCC Inc.) has 8.60 kb size with markers ampR, G418R, SV40 promoter, pBR322 early promoter, and pMB1 replicon. Firstly, primary cells were cultured in 75 cm^2^-flasks, and once these flasks became confluent, cells were dissociated using 0.05% trypsin. An average of 7×10^6^ cells in DMEM medium containing 10% FCS were transfected with 30 microg pSV3 plasmid by electroporation using Gene Pulser Xcell Electroporation Systems (Biorad Inc.). Cuvettes of 4 mm were used for these experiments. Approximately 4–6 weeks were taken to obtaining initial clones of SV40 transformed cells.

### ASA assays in 384-well microplates

The 384-well white solid-bottom plates containing cultured SV40t cells were utilized in the fluorescence-quench absorbance ASA assays. In the initial experiments, concomitant 384-well black clear-bottom plates were used to measure absorbance at 515 nm in Safire^2^ TECAN plate reader. Cells were initially cultured to confluence in a 75 cm^2^-flask and then dissociated with 0.05% of trypsin, diluted in 10% FCS DMEM (non-phenol red) medium to reach a concentration of ∼2×10^3^ cells/microL. Twenty microL were transferred per well in 384-well plates, which were then kept overnight in at 37°C in a tissue culture 5% CO_2_ incubator. On average 40×10^3^ cells were seeded in each well of 384-well plates. Substrate buffer was prepared with pNCS at 15 mM on the same sodium acetate buffer (0.5 M; pH 5.0) earlier described with exception of adding TX-100 (1%) to promote cell lysis. A volume of 40 microL of this substrate/lysis buffer was added per well, and plates were then incubated at room temperature for 14 hs. In order to stop the ASA enzymatic reaction, 50 microL of NaOH 1N (pH 10.5) were placed in each well. In 384-well white solid-bottom plates, fluorescence was read at excitation 525 nm and emission 595 nm using multi-well plate reader (Spectra Gemini XS). The absolute fluorescence value obtained was converted to optical density (OD), calculated as OD = −log(*sample/blank*), where *sample* represents the signal obtained from a specific well containing cells, culture medium and pNCS substrate buffer and stop reaction solution; *blank* represents the mean from signals from wells containing no cells, but only cultured medium, pNCS substrate buffer and stop reaction solution. The inter-leaved 384-well plate assays (Fig.4C and 4D) were performed as the assay guidance manual from NIH Chemical Genomics Center.

### ASA assays in 1,536-well plates

The miniaturization to 1,536-well plate was performed using 128.0/85MM, tissue culture, sterile Greiner white solid-bottom plates. Only SV40t cells were cultured in 1,536-well plates. On average 8×10^6^ cells were dispensed per well of white solid-bottom 1,536-well plates using Thermo Scientific MultiDrop Combi (∼volume of 4 microL). After 6–7 h, LOPAC compounds (46 nL) were transferred via a Kalypsys Pin-Tool equipped with a 1,536-pin array (V&P Scientific Palo Alto, CA). Substrate/lysis buffer as described above, with exception of the pNCS concentration of 1 mM, was dispensed (4 microL) after plates were incubated at 37°C (CO_2_ 5%) for 48 h. After substrate incubation for 14 h at room temperature, 2 microL of stop solution of NaOH (10 N) was added in each well and fluorescence was measured at excitation 525 nm and emission 598 nm using ViewLux (PerkinElmer). Thermo Scientific Multidrop Combi was used to dispense substrate/lysis and stop solution in 1,536-well plates in the HTS ASA assay (Table 2).

### Cell-based quantitative pilot qHTS assay

The Library of Pharmacologically Active Compounds (LOPAC), a collection of 1,280 pharmacological compounds from Sigma- Aldrich Inc. was used to perform the HTS pilot assay. One single 1,536-well plate was able to accommodate the entire LOPAC library and necessary controls as depicted in figure 5A. SV40t fibroblasts from a MLD patient (ASA-I179S) and control (ASA-WT) lines were used. Seven different concentrations of LOPAC library were chosen, ranging from 7×10^−3^to1142microM. Five-fold dilutions from the highest concentration (114.2 microM) was performed to cover a wide range of concentrations chosen based on previous qHTS assays [23]. One additional 1,536-well plate containing only dimethyl sulfoxide (DMSO) was performed as control plate. Cells were then exposed for 48 h treatment period with LOPAC compounds (one compound/well) at the seven different concentrations. After treatment, substrate/lysis buffer was dispensed in each well as above described and depicted in Table 2 and figure 5.

### Cell treatment with selected candidate small molecules

Primary cultured skin fibroblasts from control (ASA-WT) and a MLD patient (ASA-I179S) were seeded into 96-well plates. Candidate compounds presenting concentration-response curve class 2.2 and 2.4 in primary screening were tested in primary cells. These small molecules were purchased from Sigma-Aldrich Inc. (6-OH-DL-dopamine, FW; methoxyverapamil hydrochloride, FW 521.09;) and Santa Cruz Biotechnology (6-hydroxymelatonin, FW 248.28; D-erythro-Sphingosine, dihydro-, FW 301.5; I-OMe-Tyrphostin AG 538, FW 437.19; JFD00244, FW 478.54). All the compounds were diluted in dimethyl sulfoxide (DMSO) initially to reach working solutions at 18–20 mM concentrations. Cells were grown to confluence in 96-well plate and then treated in 5 concentrations: 7.1, 14.3, 28.6, 57.1 and 114.2 microM in DMEM with FCS at 10% for 5 days. After five days, culture medium was removed and ASA assays were performed in 96-well plates (where cells were cultured) using colorimetric pNCS. Absorbance was measured at 515 nm as described in Supporting Information S1.

### Other lysosomal enzyme assays

Measurements of total beta-hexosaminidase (Hex) and beta-galactosidase activity were performed using cultured cell lysates as previously described [6].

### NBD-sulfatide and microscopy assays

Firstly, *N*BD-sulfatide was conjugated with fatty acid free human albumin as previously described [18]. Fibroblast cells were grown on 6-well plate over a coverslip to 70% confluence. Culture medium was changed to DMEM without FCS but containing 10 nmoles of albumin-conjugated *N*BD-sulfatide for 24 h incubation at 37°C and 5%CO_2_. Cells were then washed with PBS five times. Coverslips with cells were then mounted into glass slides with antifade solution from Prolong Gold (Invitrogen Inc.). Confocal laser scanning microscopy on the Zeiss LSM 510 confocal system was performed. All the images were taken using 40 and 60 X 1.4 numerical apertures with Apochromat objectives. Images were performed using the Zeiss 2009 software.

### Statistics

Where applicable, data are expressed as the mean ± standard deviation (S.D.). Comparisons of parametric data were analyzed in the use of conventional parametric statistical methods as two-tail Student's *t* test. The statistical test Z' factor was used to measure the quality of the assay and its applicability to high throughput screening [40]. Assays with Z' factor of 0.5 indicate that the assay robust enough to identify enhancement of enzyme activity reliably [40]. Coefficient of variance (CV) and signal-to-noise ratios were also calculated when comparing signals from ASA activity from wild type (WT) and mutant (MUT) cell lines. Concentration curve analysis was performed utilizing non-linear regression curve fit using Prism version 5.0d.

## Supporting Information

Supporting Information S1 (DOCX)

Figure S1 **ASA throughput assay in 96-well plates.** ASA assays were performed initially in primary skin fibroblast cultured in 96-well plates. (**A**) In a control primary skin fibroblasts (ASA-WT), substrate pNCS concentrations were assessed. After reaching full confluence, cells were exposed to cell lysis solution (*Methods*), before substrate solution (pNCS 10 mM) was added. Blue bars represent wells assayed (4/substrate concentration) and white bars, the correspond blanks containing wells with cells treated the same way but loaded stop solution before substrate buffer. (**B**) In the plate uniformity assessment (inter-leaved signal format), ASA activity was measured using pNCS (10 mM) from control fibroblasts with ASA-WT (squares – maximum signal) and the MLD patient fibroblasts with ASA-I179S (triangles – mid signal) cultured in three 96-well plates, which were assayed in different days. Background signal (minimum signal) was derived from wells with cells but as previously the stop solution was added before substrate solution (diamonds). The mean signal-to-noise ratio was 4.99+0.66 and 2.25+0.15 for the control (squares) and I179S mutant (triangles) ASA cell lines, respectively. (**C**) DMSO tolerance test showed that concentrations over 2% in the reaction assay volume decreases the ASA activity against pNCS. (**D**) Performing similar assays in 96-well plate, SV40-transformed fibroblast from control (SV40t –CTRL) and MLD patient cells (SV40t-MLD) showed increased absorbance signals correspondent primary (prim) cell lines: prim-CTRL and prim-MLD. MLD patient cell line tested carries mutation ASA-c.459+1G>A/E482G, resulting in a lower ASA residual activity than ASA-I179S.
(TIF)

Figure S2 **Scatter plot from the quantitative cell-based HTS assay for ASA using LOPAC.** Panels represent SV40t cells from control (ASA-WT) and MLD patient (ASA-I179S). SV40t MLD patient cells were exposed to the 1,280 compounds from LOPAC in different concentrations (panels **A–E**). OD signal derived from MLD patient cells treated with LOPAC were located in columns 5–44. Columns 2, 3, 46 and 47 represent OD signals from wells contained control cells, which were not treated with compounds. MLD patient fibroblasts treated only with DMSO were located in columns 4 and 45. The small molecule concentrations MLD patient cells were exposed to: 36×10^−3^ microM (**A**), 0.18 microM (**B**), 0.9 microM (**C**), 4.5 microM (**D**) and 22.8 microM (**E**). Scatter plot results from cells seeded in the same manner but treated with DMSO and the highest (114.2 microM) and lowest (7×10^−3^ microM) concentrations of LOPAC are shown in Figure 6 (article).
(TIF)

Table S1 **Curve classification of concentration-response curves.**
(DOCX)

Table S2 **Cell viability assay for treatment of candidate compounds from HTS.**
(DOCX)
