# Supplementary material for: Novel Patient Cell-Based HTS Assay for Identification of Small Molecules for a Lysosomal Storage Disease
Source: PLoS One. 2011 Dec 21;6(12):e29504. doi: 10.1371/journal.pone.0029504 (PMC3244463; doi:10.1371/journal.pone.0029504)
Supplement: Supporting Information S1 — (DOCX) [file pone.0029504.s001.docx]

**Novel Patient Cell-Based HTS Assay for Identification of**

**Small Molecules for a Lysosomal Storage Disease**

Haifeng Geng^1*^, Grace Whiteley^3*^, Jameson Ribbens^1^, Wei Zheng^3^, Noel Southall^3^, Xin Hu^3^, Juan J. Marugan^3^, Marc Ferrer^3^, Gustavo H.B. Maegawa^1,2^

^1^McKusick-Nathans Institute of Genetic Medicine, ^2^Department of Pediatrics, Johns Hopkins University School of Medicine, Baltimore, MD 21205, United States

^3^National Institutes of Health, NIH Chemical Genomics Center, Rockville, MD 20850, United States

**Equal contribution*

**SUPPORTING INFORMATION**

**Initial 96-well miniaturization of ASA assay using primary cultured fibroblast lines**

pNCS is the standard and traditional substrate used for the biochemical diagnosis of MLD [19]. Despite being a colorimetric substrate, which is less ideal for throughput assays, pNCS showed an encouraging assay window in 96- well plates (Fig.S1). A residual activity of ASA was detectable at a low level using a fibroblasts from a late onset MLD patient with mutations I179S/R244C in *ARSA* gene [1]. Since R244C is a null *ARSA* mutation associated with a severe phenotype [2], detectable ASA residual activity is derived from I179S mutant ASA, which is commonly found in late onset forms of MLD [30].

Using primary fibroblasts from controls (ASA-WT) and MLD patients (ASA-I179S), initial assays were performed in 96-well plates. Plate-uniformity and signal variability assessment was performed to generate initial statistical parameters for the colorimetric pNCS assay. Three 96-well plates each combining layouts of control (ASA-WT) and MLD patient (ASA-I179S) fibroblasts were cultured to confluence and assayed using 10 mM pNCS. Incubation for 14h-period at room temperature allowed complete cell lysis, and consequently less signal variation was observed. The coefficients of variation (CV) were 1.23% and 0.62% in control and MLD patient (ASA-I179S) primary fibroblasts, respectively (Fig.S2). The CV of background signal was 0.55%. Taking the spectophotometric signal measured from the background as minimal, and the signal from control cell lines as maximal, a statistical Z’ paramater was calculated resulting in value of 0.5 [40].

**SUPPLEMENTAL EXPERIMENTAL PROCEDURES**

**ASA assays in cell lysates and 96-well plates**

Three different assays were used to measure ASA enzymatic activity in cells from MLD patients and controls. All of these assays were variations of the modified Baum assay [19] to selectively measure ASA activity and minimize signal from ASB by increasing the concentration of NaCl (1.7 M) in the assay buffer [20]. Fifteen microL of cultured cell lysates were diluted to 100 microL in sodium acetate buffer (0.5 M; pH 5) containing 0.5% human serum albumin in a borisilicate tube. Substrate buffer stock was prepared in sodium acetate buffer (0.5M, pH 5) containing pNCS (10 mM) along with NaCl (1.7 M) and Na_2_P_2_O_7_ (0.5 mM) [19]. Two hundred microL of substrate buffer were added to the sample (pNCS final concentration of 6.64 mM). The assay was performed in a 37^o^C water bath for 120 min. The reaction was stopped with 200 microL of NaOH (1 N). A fraction of each tube (200 microL) was transferred to a well of 96-well plate to measure trans-absorbance at 515 nm using a spectrophotometric plate reader (SpectraMAX 190; Molecular Devices TM). Specific protein concentrations from cell lysates were determined by Bradford protein assay. For initial ASA assay in microplates, fibroblasts were cultured in 96-well plates until total confluence. After removing culture medium from wells, cells were washed twice with PBS, and 28 microL of lysis solution (MPER, Thermo Fisher) containing protease inhibitors was added per well. After keeping the plate in 4^o^C for 120 min, for cell lysis, plate was then equilibrated at room temperature and 100 microL of 10 mM pNCS substrate solution, prepared as described above, were added in each well. After 14 h incubation in room temperature, the reaction was terminated using NaOH (1N) and the absorbance signal was measured in a multiplate spectophotometer (SpectraMAX 190) at 515 nm.

**Cytotoxicity assay**

When testing some of the small molecules, which presented curve class 2.2 and 2.4, a cell viability assay was performed in 96-well plate utilizing CellQuant-Blue^TM^ reagents following recommended protocol. This is a non-radioactive fluorescent assay is available by BioAssay Systems.

**SUPPLEMENTAL REFERENCES**

1. Fluharty AL, Fluharty CB, Bohne W, von Figura K, Gieselmann V (1991) Two new arylsulfatase A (ARSA) mutations in a juvenile metachromatic leukodystrophy (MLD) patient. Am J Hum Genet 49: 1340-1350.

2. Draghia R, Letourneur F, Drugan C, Manicom J, Blanchot C, et al. (1997) Metachromatic leukodystrophy: identification of the first deletion in exon 1 and of nine novel point mutations in the arylsulfatase A gene. Hum Mutat 9: 234-242.
