# Supplementary figures and images for: Novel Patient Cell-Based HTS Assay for Identification of Small Molecules for a Lysosomal Storage Disease
Source: PLoS One. 2011 Dec 21;6(12):e29504. doi: 10.1371/journal.pone.0029504 (PMC3244463; doi:10.1371/journal.pone.0029504)

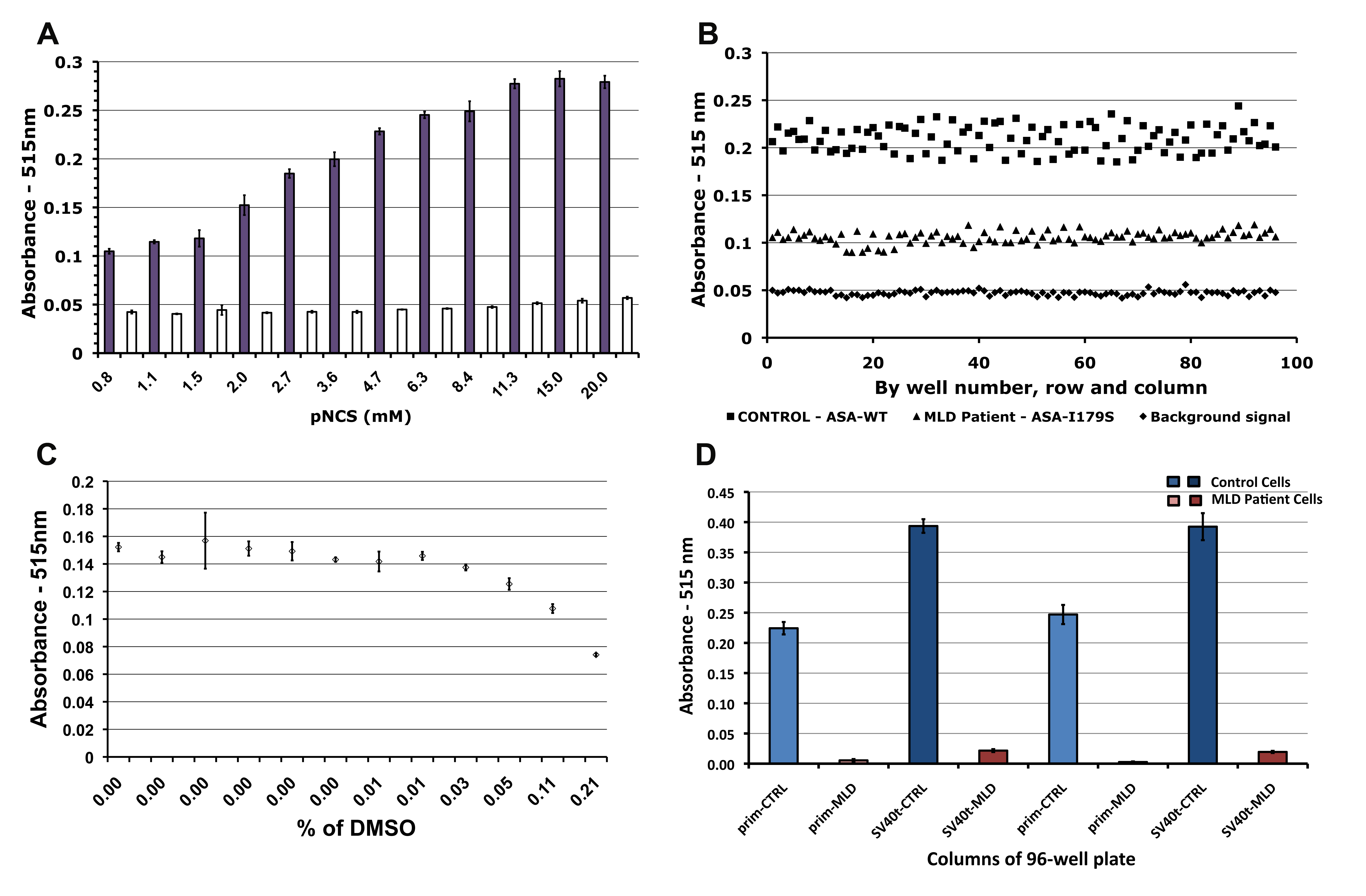

Supplement: Figure S1 — ASA throughput assay in 96-well plates. ASA assays were performed initially in primary skin fibroblast cultured in 96-well plates. (A) In a control primary skin fibroblasts (ASA-WT), substrate pNCS concentrations were assessed. After reaching full confluence, cells were exposed to cell lysis solution (Methods), before substrate solution (pNCS 10 mM) was added. Blue bars represent wells assayed (4/substrate concentration) and white bars, the correspond blanks containing wells with cells treated the same way but loaded stop solution before substrate buffer. (B) In the plate uniformity assessment (inter-leaved signal format), ASA activity was measured using pNCS (10 mM) from control fibroblasts with ASA-WT (squares – maximum signal) and the MLD patient fibroblasts with ASA-I179S (triangles – mid signal) cultured in three 96-well plates, which were assayed in different days. Background signal (minimum signal) was derived from wells with cells but as previously the stop solution was added before substrate solution (diamonds). The mean signal-to-noise ratio was 4.99+0.66 and 2.25+0.15 for the control (squares) and I179S mutant (triangles) ASA cell lines, respectively. (C) DMSO tolerance test showed that concentrations over 2% in the reaction assay volume decreases the ASA activity against pNCS. (D) Performing similar assays in 96-well plate, SV40-transformed fibroblast from control (SV40t –CTRL) and MLD patient cells (SV40t-MLD) showed increased absorbance signals correspondent primary (prim) cell lines: prim-CTRL and prim-MLD. MLD patient cell line tested carries mutation ASA-c.459+1G>A/E482G, resulting in a lower ASA residual activity than ASA-I179S. (TIF) [file pone.0029504.s002.tif]

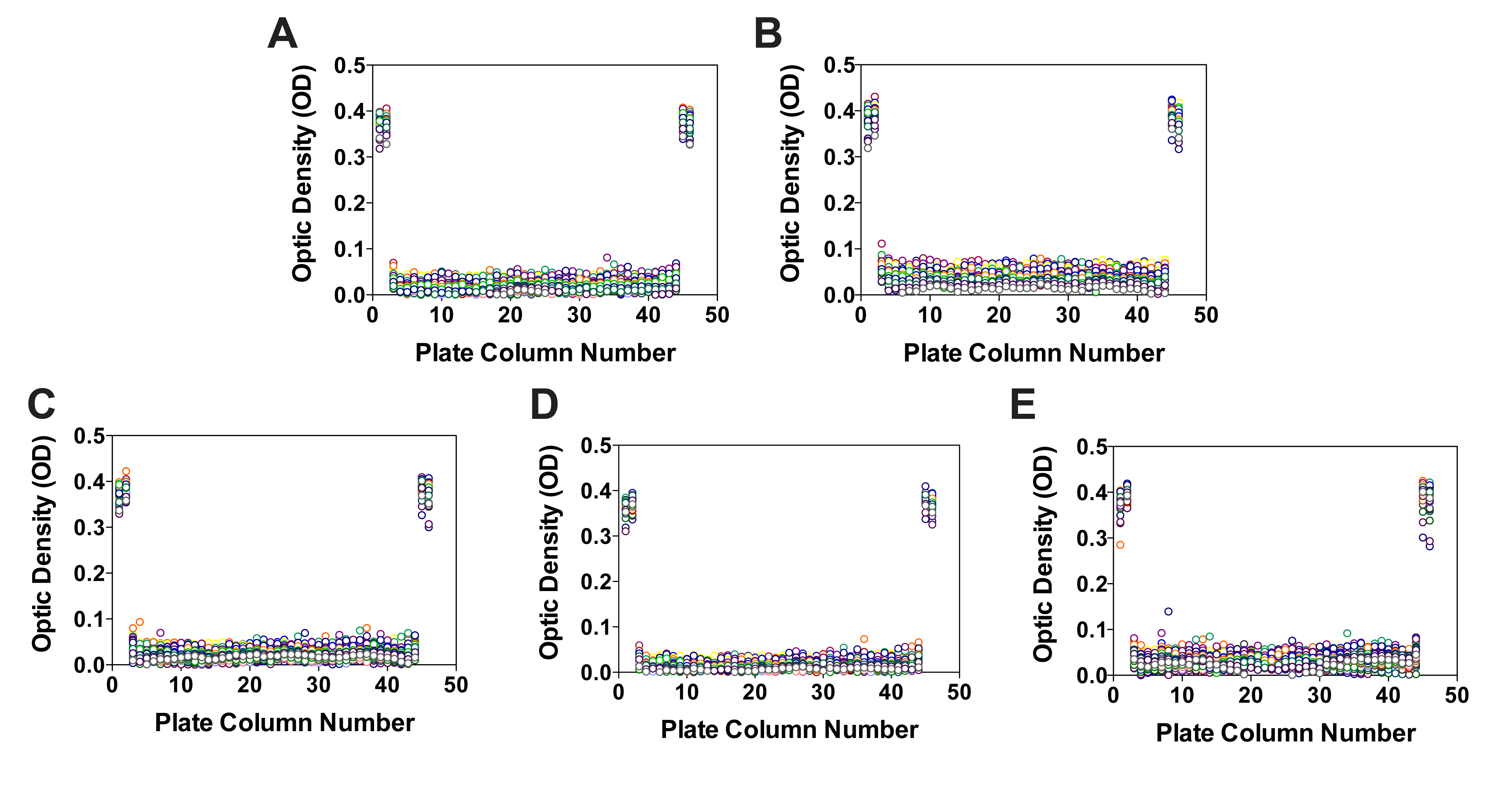

Supplement: Figure S2 — Scatter plot from the quantitative cell-based HTS assay for ASA using LOPAC. Panels represent SV40t cells from control (ASA-WT) and MLD patient (ASA-I179S). SV40t MLD patient cells were exposed to the 1,280 compounds from LOPAC in different concentrations (panels A–E). OD signal derived from MLD patient cells treated with LOPAC were located in columns 5–44. Columns 2, 3, 46 and 47 represent OD signals from wells contained control cells, which were not treated with compounds. MLD patient fibroblasts treated only with DMSO were located in columns 4 and 45. The small molecule concentrations MLD patient cells were exposed to: 36×10−3 microM (A), 0.18 microM (B), 0.9 microM (C), 4.5 microM (D) and 22.8 microM (E). Scatter plot results from cells seeded in the same manner but treated with DMSO and the highest (114.2 microM) and lowest (7×10^−3^ microM) concentrations of LOPAC are shown in Figure 6 (article). (TIF) [file pone.0029504.s003.tif]
