# Supplementary material for: Novel Patient Cell-Based HTS Assay for Identification of Small Molecules for a Lysosomal Storage Disease
Source: PLoS One. 2011 Dec 21;6(12):e29504. doi: 10.1371/journal.pone.0029504 (PMC3244463; doi:10.1371/journal.pone.0029504)
Supplement: Table S1 — Curve classification of concentration-response curves. (DOCX) [file pone.0029504.s004.docx]

**Table S1. Curve classification of concentration-response curves**

| **Curve Class** | **Asymptotes** | ***r^2^*** | **Efficacy** | **Description** |
| --- | --- | --- | --- | --- |
| **1.1** | **2**  **(higher and lower)** | **>** 0.9 | >80% | Complete curve; good fit; high efficacy |
| **1.2** |  |  | Min - 80% | Complete curve; good fit; partial efficacy |
| **1.3** |  | **<** 0.9 | >80% | Complete curve; poor fit; high efficacy |
| **1.4** |  |  | Min - 80% | Complete curve; poor fit; partial efficacy |
| **2.1** | **1 (lower)** | **>** 0.9 | >80% | Partial curve; good fit; high efficacy |
| **2.2** |  |  | Min - 80% | Partial curve; good fit; partial efficacy |
| **2.3** |  | **<** 0.9 | >80% | Partial curve; poor fit; high efficacy |
| **2.4** |  |  | Min - 80% | Partial curve; poor fit; partial efficacy |
| **3** | **1 (lower)** |  | > Min. | Single point of activity |
| **4** | **None** |  | < Min | Inactive |
| **5** | **None** |  | < Min | Inconclusive |

* Classification as published in Inglese *et al.* 2006 (23). Min, minimal.
