# Supplementary material for: Novel Patient Cell-Based HTS Assay for Identification of Small Molecules for a Lysosomal Storage Disease
Source: PLoS One. 2011 Dec 21;6(12):e29504. doi: 10.1371/journal.pone.0029504 (PMC3244463; doi:10.1371/journal.pone.0029504)
Supplement: Table S2 — Cell viability assay for treatment of candidate compounds from HTS. (DOCX) [file pone.0029504.s005.docx]

**Table S2. Cell viability assay for treatment of candidate compounds from HTS.**

|  | **Cell Viability (%)** | | | | | | | | | |
| --- | --- | --- | --- | --- | --- | --- | --- | --- | --- | --- |
| ***Concentration (microM)*** | ***7.1*** | | ***14.3*** | | ***28.6*** | | ***57.1*** | | ***114.2*** | |
| ***Cell Lines*** | ***CTRL*** | ***MLD*** | ***CTRL*** | ***MLD*** | ***CTRL*** | ***MLD*** | ***CTRL*** | ***MLD*** | ***CTRL*** | ***MLD*** |
| ***Small Molecules from LOPAC**** | | | | | | | | | | |
| ***JFD00244*** | 61.1 | 75 | 27 | 9.5 | 28.1 | 19.5 | 2.9 | 1 | 3.7 | 0.8 |
| ***6-OH-DL-Dopa*** | 112 | 100 | 102.6 | 103.2 | 107.2 | 95.2 | 99.4 | 92.1 | 109.3 | 109.6 |
| ***Methoxyverapamil HCl*** | 78.8 | 102.5 | 76.2 | 105.3 | 73.2 | 96 | 76.8 | 84.6 | 83.7 | 78.1 |
| ***DL-E-Dihydrosphingosine*** | 69 | 97.7 | 25.9 | 76 | 0.5 | 0.6 | 0.9 | 0.6 | 0.5 | 0.7 |
| ***6-OH-melatonin*** | 90.1 | 84.7 | 90.7 | 78.4 | 80.7 | 75 | 89.6 | 95 | 88.4 | 88.6 |
| ***Tyrphostin AG 536*** | 79.6 | 102.3 | 88.3 | 97.5 | 69.2 | 92.6 | 58.3 | 91.7 | 79.5 | 74.6 |

*LOPAC, Library of Pharmacological Active Compounds.
